# A Dynamic and Effective Peptide-Based Strategy for Promptly Addressing Emerging SARS-CoV-2 Variants of Concern

**DOI:** 10.3390/ph17070891

**Published:** 2024-07-04

**Authors:** Michela Murdocca, Isabella Romeo, Gennaro Citro, Andrea Latini, Federica Centofanti, Antonella Bugatti, Francesca Caccuri, Arnaldo Caruso, Francesco Ortuso, Stefano Alcaro, Federica Sangiuolo, Giuseppe Novelli

**Affiliations:** 1 Department of Biomedicine and Prevention, University of Rome Tor Vergata, Via Montpellier 1, 00133 Rome, Italy; michela.murdocca@uniroma2.it (M.M.); gennaro.citro46@gmail.com (G.C.); a.latini@med.uniroma2.it (A.L.); federica.centofanti@gmail.com (F.C.); novelli@med.uniroma2.it (G.N.); 2 Dipartimento di Scienze della Salute, Università “Magna Græcia” di Catanzaro, Campus “S. Venuta”, Viale Europa, 88100 Catanzaro, Italy; isabella.romeo@unicz.it (I.R.); ortuso@unicz.it (F.O.); alcaro@unicz.it (S.A.); 3 Net4Science Srl Academic Spin-Off, Università “Magna Græcia” di Catanzaro, Campus “S. Venuta”, Viale Europa, 88100 Catanzaro, Italy; 4 Section of Microbiology, Department of Molecular and Translational Medicine, University of Brescia, 25123 Brescia, Italy; antonella.bugatti@unibs.it (A.B.); francesca.caccuri@unibs.it (F.C.); caruso@med.unibs.it (A.C.); 5 IRCCS Neuromed Mediterranean Neurological Institute, 86077 Pozzilli, Italy; 6 Department of Pharmacology, School of Medicine, University of Nevada, Reno, NV 89557, USA

**Keywords:** peptides, protein–protein interaction (PPI), SARS-CoV-2, surface plasmon resonance, RBD variants, pseudovirus

## Abstract

Genomic surveillance based on sequencing the entire genetic code of SARS-CoV-2 involves monitoring and studying genetic changes and variations in disease-causing organisms such as viruses and bacteria. By tracing the virus, it is possible to prevent epidemic spread in the community, ensuring a ‘precision public health’ strategy. A peptide-based design was applied to provide an efficacious strategy that is able to counteract any emerging viral variant of concern dynamically and promptly to affect the outcomes of a pandemic at an early stage while waiting for the production of the anti-variant-specific vaccine, which require longer times. The inhibition of the interaction between the receptor-binding domain (RBD) of the severe acute respiratory syndrome coronavirus 2 (SARS-CoV-2) and one of the cellular receptors (DPP4) that its receptors routinely bind to infect human cells is an intriguing therapeutic approach to prevent the virus from entering human cells. Among the other modalities developed for this purpose, peptides surely offer unique advantages, including ease of synthesis, serum stability, low immunogenicity and toxicity, and small production and distribution chain costs. Here, we obtained a potent new inhibitor based on the rearrangement of a previously identified peptide that has been rationally designed on a cell dipeptidyl peptidase 4 (DPP4) sequence, a ubiquitous membrane protein known to bind the RBD-SPIKE domain of the virus. This novel peptide (named *DPP4-derived*), conceived as an endogenous “drug”, is capable of targeting the latest tested variants with a high affinity, reducing the VSV* DG-Fluc pseudovirus Omicron’s infection capacity by up to 14%, as revealed by in vitro testing in human Calu-3 cells. Surface plasmon resonance (SPR) confirmed the binding affinity of the new *DPP4-derived* peptide with Omicron variant RBD.

## 1. Introduction

A few months ago, the head of the UN World Health Organization (WHO) declared the end of the COVID-19 public health emergency [1]. It is important to stress that this declaration does not mean the disease is no longer a global threat. Full eradication of COVID-19 is not on the horizon due to the emergence of new variants of concern and the global administration of COVID-19 vaccines, which have significantly altered the clinical landscape of the disease. As the SARS-CoV-2 pandemic is a novel and contemporary disease, its invasive properties have not been thoroughly explored or fully understood [2]. A substantial amount of research has been conducted within a relatively short period to enhance our understanding of the structure of SARS-CoV-2 [3]. However, we have gathered substantial information on the dynamics of viral evolution and how these dynamics shape interactions with the host. This research aspect remains fundamental for the prevention, protection, and management of this disease, particularly for patients at high risk of severe illness [4].

SARS-CoV-2 is a single-stranded RNA β Coronavirus, and despite its size, its mutation rate is lower than that of other RNA viruses [5,6,7]. In November 2021, the WHO identified the Omicron variant of SARS-CoV-2, known as B.1.1.529, as a variant of concern (VOC). By early 2022, the Omicron variant and its five lineages (i.e., BA.1, BA.2, BA.3, BA.4, BA.5, and XBB) had become the primary cause of COVID-19 worldwide. The Omicron XBB.1.5 subvariant is a sublineage of the XBB variant, resulting from the recombination of two BA.2 sublineages. In January 2023, a rapid increase in the prevalence of the XBB.1.5 subvariant led to it causing 49.1% of COVID-19 cases in the US. This increase was attributed to immune evasion from prior infection or vaccination, mutations like F486P having arisen within the spike protein, and an increased affinity for the ACE2 receptor that binds SARS-CoV-2. Additionally, current booster vaccines may not provide sufficient protection against infection by this subvariant, which has been colloquially referred to as the ‘Kraken’ subvariant. With the expansion of worldwide genome sequencing surveillance, an increase in the number of newly identified variants is anticipated. Vigilant monitoring of all newly identified variants is essential to enable the implementation of necessary countermeasures as early as possible. Specific mutations in the proteins of SARS-CoV-2 can potentially compromise the effectiveness of drugs, representing a significant cause for concern. Computational techniques are indispensable for monitoring and understanding the continuous mutations in viruses like SARS-CoV-2 [8,9,10]. They enable researchers to track the virus’s evolution and evaluate the impact of binding of potential inhibitors to the receptor.

With the knowledge and experience gained from the COVID-19 pandemic, there is an urgent need to develop novel strategies for quickly predicting and preventing the emergence and transmission of novel pathogens. Clinical trials are underway, testing potential antiviral therapeutic targets, such as inhibiting the viral enzymes responsible for genome replication or blocking viral entry into human cells. Among the potential approaches to pharmacologically combat COVID-19 (including small-molecule drugs, interferon therapies, vaccines, oligonucleotides, and monoclonal antibodies), in the future, peptides may play various roles in the prevention and management of SARS-CoV-2 [11,12,13,14,15].

It is now well established that SARS-CoV-2 uses angiotensin-converting enzyme 2 (ACE2), the same receptor as SARS-CoV, to infect humans [16]. However, other studies have shown that SARS-CoV-2 also binds to human dipeptidyl peptidase (DPP4/CD26) when entering cells in the respiratory tract. The interaction between the SARS-CoV-2 spike glycoprotein and DPP4 is a key factor for virulence [17,18]. Interestingly, another recent study has indeed reported a correlation between DPP4 and ACE2, indicating the relevance of both membrane proteins in the pathogenesis of virus entry [19]. Consequently, we directed our focus toward this less-explored aspect, investigating the potential use of DPP4 peptides as an innovative approach in the pharmacologic treatment of COVID-19. In our previous work, the primary mechanism of the peptide was to block the S protein’s receptor-binding region from interacting with DPP4, thus acting preventively against infection. Subsequently, a peptide bearing the DPP4 sequence (DPP4_270–295_) was designed and used in vitro to assess its capacity to block VSV* DG-Fluc pseudovirus infection [19]. In particular, the DPP4_270–295_ peptide exhibited a higher inhibitory capacity against both WT and its variant particles (i.e., D614G, B1.1.7, B1.351, and P1), circulating during 2021 until *Omicron B.1.1.529* [19]. The higher inhibitory capacity was nonetheless starting to reduce with the new variants of concern occurring. Therefore, at this point, we decided to rationally design a novel peptide, named *DPP4-derived*, that is able to prevent the entry of the virus into the host cell. We accomplished this by using a 3D mapping approach and docking simulations. This was done to adapt the novel rearranged peptide for enhancing its inhibitory potential against other variants of concern, such as BA4.5, B.Q.1.1, and XBB.1.5, which was no longer achieved by the native DPP4_270–295_ peptide. One of these designed peptides was synthesized and tested in vitro, revealing its effective capacity of inhibiting SARS-CoV-2 infection up to the latest Omicron subvariant, which is still spreading. Moreover, proinflammatory chemokine and cytokine expression levels were reduced. A surface plasmon resonance (SPR) assay was conducted to investigate the interaction between the *DPP4-derived* peptide and the RBD of SARS-CoV-2 from Omicron sublineages in comparison to that of native DPP4_270–295_. Due to its good binding to RBD and its inhibitory properties, *DPP4-derived* peptide could serve as a promising lead candidate for the development of prophylactic or therapeutic treatments against SARS-CoV-2. Thus, our data indicate that peptide technology offers a strategy that can dynamically and promptly react to any new viral variant of viral infection, since the peptide sequence can be easily tailored in silico to combat them effectively. The reaction of the polypeptide with the key molecule of the pathogens inhibits and/or reduces the infecting capacity, with effects on the concentration and speed of diffusion, pending the production of a vaccine. Thus, while waiting for the definitive vaccine, which is difficult to obtain with the sudden onset of new variants, the use of peptides could temporarily stop the viral SARS-CoV-2 infection.

## 2. Results

### 2.1. In Silico Studies of the DPP4_270–295_ Peptide against the RBD of Emenging Variants of Concern

**Figure 1:**
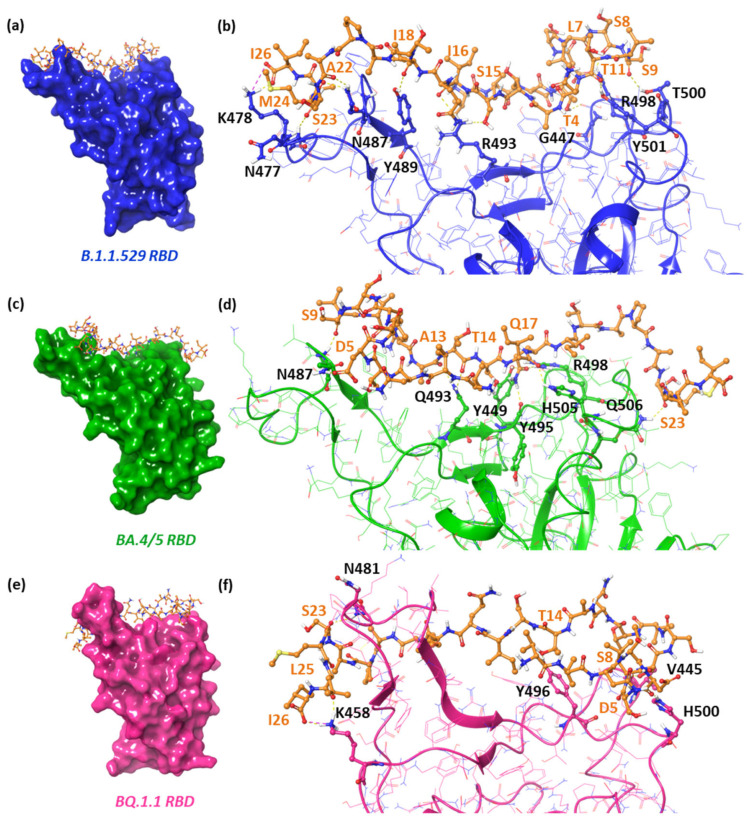
Left-hand images show the zoomed-in context of the DPP_4270–295_ peptide in complex to (**a**) B.1.1.529, (**c**) BA.4/5, and (**e**) BQ.1.1 RBD. The key contacting elements inside include (**b**) the B.1.1.529 RBD/DPP4_270–295_ conformation, (**d**) BA.4/5 RBD/DPP4_270–295_, and (**f**) BQ.1.1 RBD/DPP4_270–295_. All amino acids involved in the interaction are represented as balls and sticks, while the H-bonds and salt bridges are shown as yellow and purple dashed lines, respectively.

To study the theoretical binding affinity of the DPP4_270–295_ peptide against the RBD pocket of the newest variants, we performed molecular recognition by using High Ambiguity Driven protein–protein DOCKing (HADDOCK) ver. 2.4. As reported in Figure 1, it was found that DPP4_270–295_ established several H-bonds with the N477, K478, N487, Y489, R493, R498, T500, and Y501 residues placed into the B.1.1.529 RBD, with a total buried surface area (BSA) of 1577.5 ± 35.4 and a HADDOCK score equal to −80.6 (Table 1). 

Concerning the BA.4/5 RBD pocket, we observed that DPP4_270–295_ was able to interact with Y449, N487, Q493, Y495, R498, H505, and Q506 via H-bonds. Finally, analyzing the interactions between DPP4_270–295_ and the BQ.1.1 variant, the number of H-bonds was significantly reduced, only involving V445, K458, N481, Y496, and H500 residues. 

### 2.2. DPP4_270–295_ Peptide Partially Blocks SARS-CoV-2 Entry into Cells

**Figure 2:**
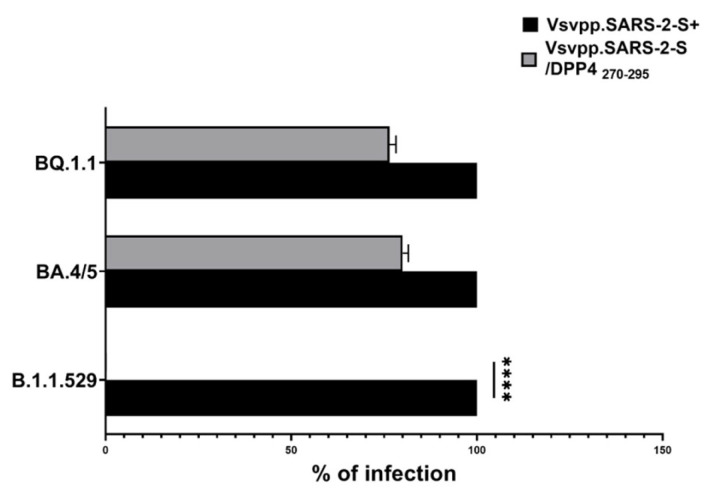
Vero E6 cells were inoculated with pseudotype particles bearing the S proteins of the indicated Vsvpp.SARS-2-S Omicron variants (B.1.1.529, BA.4/5, and BQ.1.1), together with DPP4_270–295_. Infection efficiency was quantified by measuring virus-encoded luciferase activity in cell lysates at 48 h post-transduction, expressed as a percentage (Vsvpp.SARS-2/DPP4_270–295_; grey bars), and normalized with respect to Omicron variants (Vsvpp.SARS-2-S^+^) alone (black bars). The data presented are the average of three biological replicates. Error bars indicate the standard deviation ± standard error of the mean (SEM). **** *p* < 0.0001 based on a one-way ANOVA test.

In our previous results [19], we demonstrated that DPP4_270–295_ acted in order to mask the virus to intercept the DPP4 receptor and other known receptors on target cells. Moreover, during our experiments, novel variants of COVID-19 (i.e., Omicron *B.1.1.529,* BA.4/5, and BQ.1.1.) occurred, causing increased infectiousness and virulence. Considering this, to verify the efficiency, DPP4_270–295_ peptide was used at the same concentration tested previously, i.e, 100 µg (18), without observing any toxic effect. We infected cells by using a recombinant vesicular stomatitis virus (rVSV) carrying the spike protein of SARS-CoV-2 (GenBank:MN908947) variants (Omicron *B.1.1.529*, *BA.4/5*, *BQ.1.1)*. Specifically, we first infected Vero E6 cells, and after 48 h, we performed a luciferase assay to test the efficiency of virus entry. Each obtained value was normalized with respect to *VSV_Pseudo SARS-CoV-2 (Vsvpp.SARS-2-S^+^)*. As observed in Figure 2, the DPP4_270–295_ peptide showed a high capacity to inhibit the Omicron *B.1.1.529* entry (**** *p* <0.0001), while, when using pseudotypes Omicron *BA.4/5* and *BQ.1.1*, the DPP4_270–295_ peptide reduced virus entry into target cells only by 20% and 23.3%, respectively. 

### 2.3. Design of Novel DPP4-Derived Binder of SARS-CoV-2 RBD Domain

**Figure 3:**
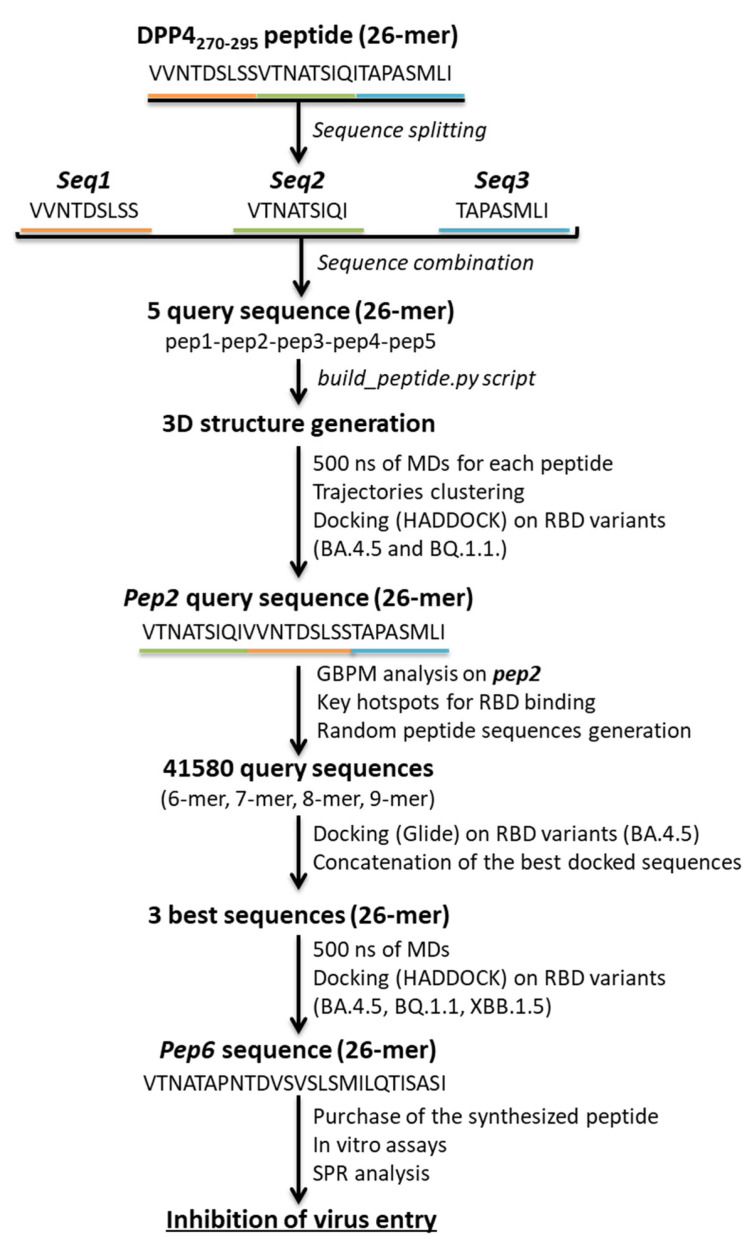
Workflow for the design of novel *DPP4-derived* peptides against SARS-CoV-2 RBD domain of emerging variants.

As specific variants in the proteins of SARS-CoV-2 seem to compromise the effectiveness of the DPP4 peptide, as other drugs, computational techniques are indispensable for monitoring and understanding the continuous variations. Here, we designed a series of novel *DPP4-derived* binders of the SARS-CoV-2 RBD domain. Based on the DPP4-binding helix (DPP4_270–295_), we split the native sequences and rearranged them into new constructs. Our goal was to optimize activity against the RBD of new variants by rearranging peptide segments. The initial splitting helped us evaluate whether the specific position of the amino acids was pivotal for the interaction with the RBD surface. Afterwards, since our strategy aimed to dynamically address a peptide against different emerging SARS-CoV-2 variants of concern, rearranging the sequence helped us to engage in interactions that the original sequence could not, enabling the design of peptides with more promising functionalities. The workflow is illustrated in Figure 3.

In detail, to narrow down the vast combinatorial search space, firstly, we split the DPP4_270–295_ peptide into three fragments that we indicated as *seq1* (VVNTDSLSS), *seq2* (VTNATSIQI), and *seq3* (TAPASMLI). Combining these fragments, we generated five query sequences (Table S1). By using the build_peptide.py script, we built the 3D structure of five *DPP4-derived* peptides, named *pep1*, *pep2*, *pep3*, *pep4,* and *pep5*. For each peptide, we submitted 500 ns of MDs, and the most representative cluster was used for docking simulations on the BA.4.5 RBD domain. Based on the docking results, it emerged that the best HADDOCK score was related to the complex *pep2*-RBD, which engaged H-bonds between V1, Q8, I9, N12, and T13 of the *pep2* sequence and V483, F490, Q493, and Y501 residues of BA.4.5 RBD, respectively. At this point, we predicted the affinity of the best peptide, such as *pep2* towards the BQ.1.1 RBD domain. Unfortunately, the worst HADDOCK score was obtained, which correlated to a reduced number of interactions (Table S2). At this stage of our strategy, a deeper analysis using GBPM was performed on DPP4_270–295_ and *pep2* in complex with the RBD of both the BA4.5 and BQ.1.1 variants. This method helps us to map the key hotspots that are responsible for PPI by combining GRID molecular interaction fields (MIFs) according to the GRAB tool algorithm [20]. BA4.5 and BQ.1.1 RBD were considered as guests, while DPP4_270–295_ and *pep2* were hosts. Three GRID probes, DRY, N1, and O, were chosen to mimic the hydrophobic, H-bond donor, and acceptor areas, respectively. The contribution of each residue was derived from the summa of its GBPM points energy in the matching frames. After calculating the total score based on all residues, the key hotspots were split into quartiles to increase the clarity for the reader. Quartile 1 (Q1) includes the residues with the most significant contributions to the interaction, decreasing until quartile 4 (Q4), which contains the residues with the weakest interactions (Table 2). 

Based on the resulting hotspot residues, we generated a total of 41,580 query sequences, combining sets of residues of different lengths (6-mer, 7-mer, 8-mer, and 9-mer) (source code is reported in the Supporting Information). By using an additional docking program, Glide, we were able to compute sequences of peptides with reduced computing times. We performed the SP-peptide docking simulations on three grid boxes that included the entire BA.4/5 RBD surface (Table S3). As a result, we concatenated the best sequences considering the amino acid superpositions, and we generated three sequences of 26-mer (Table S4). Comparing the generated sequences with the previously obtained *DPP4-derived* peptides and GBPM analysis, we directed our attention to *pep6*, thus submitting 500 ns of MDs to determine its conformational variability. The representative cluster was submitted to HADDOCK following the same protocol described above. We evaluated the theoretical binding affinity of *pep6* and the RBD domain of the BA.4/5, BQ.1.1, and XBB.1.5 variants. Remarkably, it was noticed that *pep6* showed a higher HADDOCK score in complex to the RBD of all the analyzed variants (Table 3).

**Figure 4:**
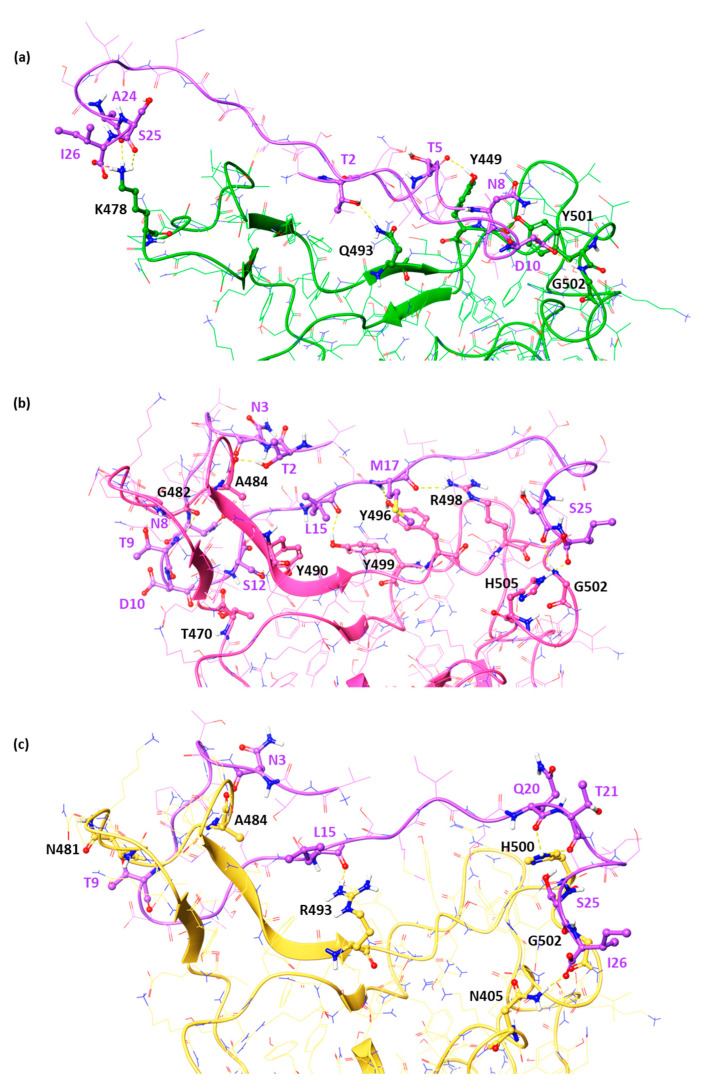
Key contacting elements inside *pep6* in complex with (**a**) BA.4/5, (**b**) BQ.1.1, and (**c**) XBB1.5 RBD. All amino acids involved in the interaction are represented as balls and sticks, while the H-bonds are shown as yellow dashed lines. BA.4/5, BQ.1.1, and XBB1.5 RBD domains are reported as green carbon, faded salmon, and yellow cartoons, respectively.

In detail, in the BA.4/5 RBD pocket, the threonines at positions 2 and 5 bound Q493 and Y449, respectively. The folding of the peptide allows it to insert into the R498-H505 loop, forming two hydrogen bonds with Y501 and G502. Finally, the presence of A24, S25, and I26 was important for stabilizing the side chain of lysine 478 through three hydrogen bonds. Regarding the two emerging variants, BQ.1.1, and XBB.1.5, comparable HADDOCK scores were observed (Table 3) for the *pep6*, equal to −91.3 and −91.7, respectively. As observed in Figure 4, this *DPP4-derived* peptide, named *pep6,* was able to interact with Y449, T470, G482, A484, Y490, Y496, R498, G502, and H505 via H-bonds in BQ.1.1 RBD. Finally, N3, T9, L15, Q20, T21, S25, and I26 engaged several H-bonds with the A484, N481, R493, H500, G502, and N405 residues of the XBB.1.5 RBD, respectively.

### 2.4. Experimental Validation of the Newly Designed Peptide pep6

**Figure 5:**
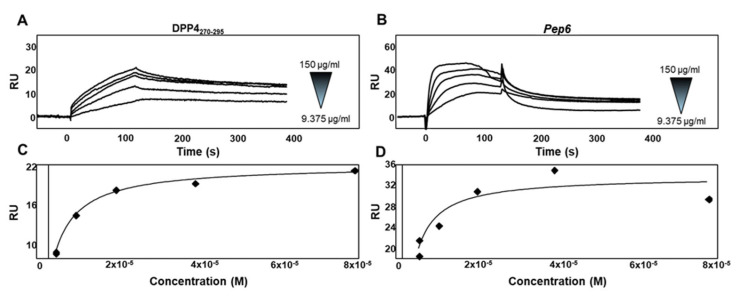
(**A**,**B**) Sensogram overlay showing the binding of increasing amounts (range from 9.375 to 150 µg/mL) of DPP4_270-295_ and *pep6* to the immobilized Omicron BA.5 RBD surface. The response, in resonance units (RU), was recorded as a function of time. (**C**,**D**) Saturation curve obtained using the values of RU bound at equilibrium from injection of increasing concentrations of free DPP4_270-295_ and *pep6* onto the immobilized Omicron BA.5 RBD sensorchip.

To verify whether *pep6* had a better interaction with the novel SARS-CoV-2 RBD variants than native DPP4_270–295_, we performed an SPR study using BIAcore technology (model software package BIAevaluation, version 3.2 Cytiva). In the SPR analysis, we used a SARS-CoV-2 Omicron BA.5 RBD, a sublineage of Omicron B.1.1.259 carrying the same amino acid mutations as XBB.1.5, which is involved in the DPP4/*pep6* interaction. To this aim, recombinant Omicron BA.5 RBD was captured onto a CM5 sensor chip. Sensograms obtained via injections of different concentrations of DPP4_270–295_ (Figure 5A) and *pep6* (Figure 5B) showed that both peptides bind to immobilized Omicron BA.5 RBD in a dose-dependent manner. The Biacore (model software package BIAevaluation, version 3.2 Cytiva) provided the kinetic parameters of the analyzed interactions. DPP4_270–295_/Omicron BA.5 RBD interactions occurred with a kinetic association constant (*k*_on_) of 1.16 × 10^3^ s^−1^ M^−1^ and a kinetic dissociation constant (*k*_off_) of 1.40 × 10^−3^ s^−1^, thus resulting in a *K_d_* value (*k*_off_/*k*_on_) equal to 1.20 × 10^−6^ M, while *pep6*/Omicron BA.5 RBD interactions occurred with a kinetic association constant of 3.07 × 10^3^ s^−1^ M^−1^ and a kinetic dissociation constant of 2.6 × 10^−3^ s^−1^, thus resulting in a *K_d_* value equal to 8.43 × 10^−7^ M. Based on the measurements of the steady-state binding levels, the affinity value at equilibrium of DPP4_270–295_/Omicron BA.5 RBD interactions (Figure 5C) was determined to be equal to 1.53 × 10^−6^ M, while *pep6* interacted with the same protein (Figure 5D) with a Kd value at equilibrium equal to 8.39 × 10^−7^ M. Taken together, these data demonstrate that *pep6* interacts with Omicron BA.5 RBD with a stronger affinity value compared to the affinity value of the native DPP4_270–295_ for the same protein.

**Figure 6:**
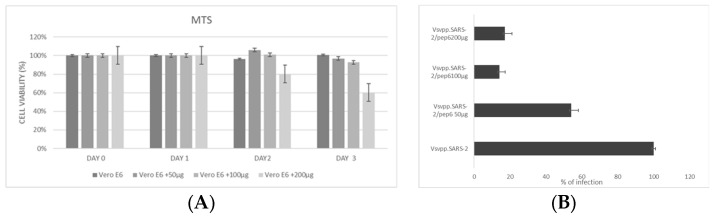
(**A**) Effects pep6 on the viability of VERO E6 cells for three days. MTS assay was performed to determine cell viability after *pep6* treatment at concentrations of 50, 100, and 200 µg for 72 h compared to untreated cells. *P*-value: VeroE6 +200 μg vs. VeroE6 at day 2 < 0.05, *p*-value VeroE6 +200 μg vs. VeroE6 at day 3 < 0.01. (**B**) Infection efficiency was quantified by measuring virus-encoded luciferase activity in Vero E6 cells infected for 48 h with VSV_Pseudo SARS-CoV-2 S Omicron XBB.1.5 alone and in combination with the synthetic peptide *pep6*. Data are expressed as percentage of infection and normalized with respect to the XBB.1.5 Omicron variant alone.

Finally, the synthetic *pep6* was employed to evaluate its ability to inhibit virus entry onto human lung epithelial cell lines that can support the propagation of SARS-CoV-2, such as Calu-3 cells, following the protocol already described. Since at the time of the study, the subvariant of Omicron XBB.1.5 was spreading, the same experiments were replicated on Calu-3 with this new strain. Previously, cell cytotoxicity was tested using the MTs reduction assay in Vero E6 cells, indicating that the tested peptide at 50 and at 100 μg showed the best concentration/toxicity ratio (Figure 6A). Also, a scale of increasing peptide concentrations was assessed (Figure 6B) for evaluating the inhibitory effects by measuring the virus-encoded luciferase activity in Vero E6. These data allowed us to select the concentration of 100 μg for the following analyses.

**Figure 7:**
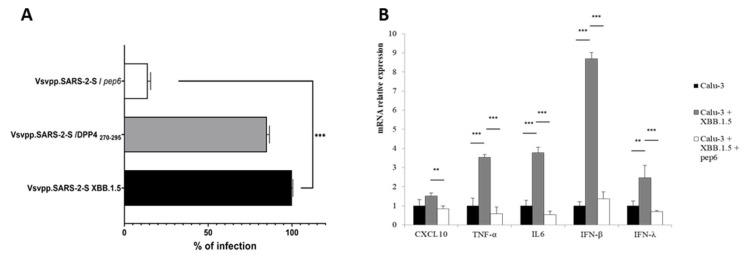
(**A**) Infection efficiency was quantified by measuring virus-encoded luciferase activity in cells infected for 48 h with VSV_Pseudo SARS-CoV-2 S Omicron XBB.1.5 alone and in combination with synthetic peptide DPP4_270–295_ and *pep6*. Data are expressed as percentage of infection and normalized with respect to the XBB.1.5 Omicron variant alone (black bar). The average data from three biological replicates are presented. Error bars indicate the standard error of the mean (±SEM). *** *p* < 0.001 based on a one-way ANOVA test. (**B**) RT-qPCR analyses of immunity-related genes in Calu-3 cells after VSV_Pseudo SARS-CoV-2 S Omicron XBB.1.5 infection and treatment with *pep6*. Data are from three independent experiments and represented as mean ± SD. ** *p* < 0.01, and *** *p* < 0.001 based on a one-way ANOVA test.

In Figure 7A, we reported that the results of the luciferase assay support the efficacy of *pep6* (at 100 μg) in reducing the infection capacity by up to 14% in a statistically significant manner (*** *p* <0.001). Vice versa, at the same concentration, DPP4_270–295_ was able to reduce the infective ability of VSV_Pseudo SARS-CoV-2 S Omicron XBB.1.5 in a negligible manner, confirming the previous data shown for Vero E6 cells (Figure 2).

Lastly, to confirm the antiviral effects of *pep6* and support its efficacy against SARS-CoV-2, we evaluated the expression of innate immune response genes. As expected, in Calu-3 cells infected with VSV_Pseudo SARS-CoV-2 S Omicron XBB.1.5, we observed that the expression levels of type I and type III IFNs and of pro-inflammatory cytokines (CXCL10, IL-6, and TNF-α) were significantly increased (Figure 7B). Interestingly, infected Calu-3 cells treated with *pep6* (white bars) showed expression levels of innate immune response genes that were comparable to the basal ones (Calu-3 cells not infected; black bars). Indeed, after *pep6* treatment, we observed a significant decrease in the mRNA levels of both classes of IFNs (*** *p* < 0.001) and of genes encoding the pro-inflammatory cytokines CXCL10 (** *p* < 0.01), IL-6 (*** *p* < 0.001), and TNF-α (*** *p* < 0.001) compared to untreated infected cells (Calu-3+XBB.1.5; grey bars). Our results suggest that *pep6* treatment is able to prevent the virus from over-activating the innate immune system.

## 3. Discussion

The global rollout of COVID-19 vaccines has played a critical role in reducing pandemic spread, disease severity, hospitalizations, and deaths. This is the time to explore the transition from the pandemic to the endemic phase. Although vaccines and drugs are enormous therapeutic advances to cure COVID-19, it is still necessary to develop new treatments. The first-generation vaccines failed to block SARS-CoV-2 infection and transmission, partially due to the limited induction of mucosal immunity and to the continuous emergence of VOCs and breakthrough infections. To meet the challenges of VOCs, limited durability, and the lack of mucosal immune response of first-generation vaccines, novel approaches are being investigated. Currently, the majority of discussed strategies are related to diagnostics and prophylactics of SARS-CoV-2 infection, which are more convenient or less limiting for the patients [21]. Unlike what has been presumed at the beginning of this ongoing pandemic, SARS-CoV-2 undergoes massive ecologic pressure (mainly host immunity) that promotes the progressive incorporation of multiple variations within its genome. Over the last two years of the pandemic, many viral variants found globally determined the first level of viral diversity, starting from the first VOC isolated in the UK in September 2020 and then moving on to Alpha (B.1.1.7), Beta (B1.351), Gamma (P.1), Delta (B.1.617.2) and Omicron (B.1.1.529), which have already undergone a process of intense mutation and recombination, generating several dozens of sublineages at the end of 2022. In fact, the currently circulating lineages, BA.1, BA.2, BA.3, BA.4, BA.5, and the predominant XBB.1.5, are mostly descendants of the Omicron variant [22,23].

Inflammatory activity still represents the main cause of all the problems resulting from the infection. Thus, preventing the entry of the virus and therefore the activation of a massive immune response could represent a successful therapeutic strategy. In addition to the ACE2 receptor, it is noteworthy that several transmembrane proteins are involved in the virus entry into the target cell. Specifically, the ability of the RBD spike to recognize the human DPP4 is a deal-breaker in exacerbating the pathogenicity of SARS-CoV-2 [24,25].

Despite their considerable therapeutic potential, the modulation of protein–protein interactions (PPIs) through inhibition or activation is difficult to reach.

Small molecules face significant challenges when attempting to target PPIs. One major issue lies in the difference in the surface area and binding characteristics between PPIs and typical small-molecule binding sites. PPIs generally feature broad and flat interfaces that span between 1500 and 3000 Å^2^, whereas small-molecule binding sites are much smaller, typically around 200 to 900 Å^2^. This mismatch means that small molecules often struggle to cover enough surface area to effectively disrupt PPIs. Another hurdle is the lack of well-defined binding pockets in many PPIs. Unlike traditional drug targets that have clear, pocket-like structures where small molecules can bind, PPI interfaces often involve amino acid residues that are spread out and oriented correctly only within the protein’s three-dimensional structure. This complexity makes it challenging for small molecules to interact in a way that disrupts the protein–protein interactions effectively. Peptides, on the other hand, present a promising middle ground. They can mimic the regions of proteins involved in PPIs more effectively than small molecules, covering larger surface areas and interacting with multiple residues simultaneously.

For this reason, there is a move towards the rational design of peptides [26]. Peptides are extensively used for the diagnostic, prevention, and therapy of certain human diseases. They can be involved as follows: (a) Vaccine development involves stimulating an immune response in the body, resulting in the production of antibodies and memory T cells. Peptide-based vaccines, such as subunit vaccines, have been used in the development of COVID-19 vaccines, like those produced by Novavax and others [27]. (b) Antiviral peptides are capable of preventing viral entry into host cells, disrupting viral replication or interfering with other essential viral processes [28]. (c) Immune modulation can potentially regulate the immune system’s response to the virus, reducing excessive inflammation and tissue damage [29,30]. (d) Diagnostic tools involve peptide-based assays, which can detect specific viral proteins or antibodies produced in response to the virus, aiding in the diagnosis of infection [31,32]. (e) Therapeutic peptides can alleviate COVID-19 symptoms and complications by targeting specific pathways involved in the disease, such as inflammation or blood clotting, in order to manage severe cases [33]. (f) Peptide libraries for drug discovery can be used to identify peptides that bind to SARS-CoV-2 proteins or interfere with viral replication, allowing researchers to discover novel antiviral compounds [34,35]. (g) Finally, human receptor binding inhibition leverages the possibility of mimicking the portions of the human receptor that are responsible for interacting with the virus, thus affecting its entry into human cells [36,37].

Regarding the prevention of COVID-19, as already known, synthetic peptides block the interactions between the virus and its main receptors, in particular against new emerging variants. Peptide-based drugs have significant benefits such as low toxicities and better specificities. In recent years, peptides have gained increased interest as candidate therapeutics, with over 80 peptide drugs presently on the market, more than 150 peptides in clinical development, and over 400 undergoing preclinical studies [37]. Indeed, peptides are easy to develop, both in terms of time and technology, and are cost-effective, which makes them good candidates for the development of hits or probes up to the proof of concept. An aspect to take into consideration is the length of the peptide that allows it to efficiently compete for the binding with the S protein and helical regions. These peptides, in comparison with low-molecular-weight drugs, show better binding due to their ability to interact with a large surface area of the protein molecule with a high selectivity and lesser toxicity [38]. A successful strategy backed by previous computational and experimental studies involves targeting the interaction between the RBD and DPP4 using linear peptides derived from the DPP4 N-terminus-binding helix [19].

In this study, our proof of concept consisted of adapting an initial peptide extracted from a segment of DPP4 and refining it through iterative redesigns to enhance its binding to the RBD. This process progressed from targeting the earliest variants to those that emerged later, ultimately optimizing its efficacy against the emerging variants of concern (VOCs). Experimental validation confirmed the effectiveness of our strategy. Starting with the DPP4 peptide (ranging from aminoacid 270 to 295) identified in our previous study [19] as being capable of interacting with the RBD of the Delta variant (B.1.617.2), we proceeded to analyze the interaction between the native DPP4_270–295_ and the emerging variants. Computational results and experimental tests demonstrated that the peptide lost affinity for the RBD of the emerging B.1.1.529, BA.4/5, and BQ.1.1 variants, resulting in reduced efficacy. For this reason, beginning with the DPP4_270–295_ sequence, in-house scripting helped us to generate amino acid sequences of different lengths based on the critical hotspot residues predicted by GBPM. Thus, the 3D mapping approach and docking simulations were employed to design and theoretically identify novel *DPP4-derived* peptides that are capable of changing specific interactions, disrupting the RBD–DPP4 interplay and counteracting the spread of incoming variants and subvariants. For the generation of the promising peptides, we considered the structures with the highest docking scores in all three grids in order to cover the entire RBD surface. The main idea in this stage is to search for the best amino acids to match with the RBD domain of two different variants by considering a large number of poses with reasonable calculation times. The main goal in the second stage was to concatenate the best fragments as a function of their amino acid orders, maintaining the same number and type of residues of the native peptide DPP4_270–295_.

Thus, among the generated *DPP4-derived* peptides, one peptide of 26-mer, named *pep6*, showed an improved theoretical binding affinity towards novel subvariants such as BA.4/5, BQ.1.1, and XBB.1.5 RBD, engaging a good network of interactions against pivotal residues. Particularly, the interactions between *pep6* and A484 and G502 resulted in common RBD of both the BQ.1.1 and XBB.1.5 variants. Despite the presence of the mutation at position 493, *pep6* retained the H-bond in the RBD of BA.4/5 and XBB.1.5. Due to this evidence, we started testing in vitro *pep6* in human lung epithelial cell lines (Calu-3 cells), demonstrating its ability to provide protection from VSV_Pseudo SARS-CoV-2 S Omicron XBB.1.5 infection. An SPR analysis also demonstrated that *pep6* directly interacts with Omicron BA.5 RBD, with a higher affinity value compared to that obtained for DPP4_270–295_ for the same variant. Moreover, to confirm the efficacy of this peptide against SARS-CoV-2, we also evaluated the expression of innate immune response-related genes in infected Calu-3 cells. While the levels of expression in the infected cells showed a noticeable fold increase, these values in *pep6*-treated cells were all comparable to baseline levels, further highlighting the peptide’s ability to block the infectious agent. These results seem to suggest that *pep6*, like other therapies that directly target the virus, prevents the over-activation of the innate immune system [39]. In summary, splitting and rearranging native peptide sequences (DPP4_270–295_) represents a powerful strategy to dynamically design a new promising peptide with enhanced theoretical affinity versus new sub-variants, as confirmed by the experimental assays. As already known, COVID-19 infection produces a multisystem condition that mainly involves the respiratory system, and in 15% of cases, leads to acute respiratory distress syndrome (ARDS), triggered mainly by elevated levels of pro-inflammatory cytokines. Interleukins, such as interleukin-6 (IL-6) and tumor necrosis factor-alpha (TNF-α), play an important role in lung damage in COVID-19 patients through impairment of the respiratory epithelium. Indeed, overproduction and uncontrolled release of pro-inflammatory molecules, defined as cytokine storms, are observed in patients who develop ARDS. Blocking SARS-CoV-2 before it activates the inflammatory cascade could therefore constitute a winning approach to prevent this serious complication of the infection.

## 4. Materials and Methods

### 4.1. Cells

The African green monkey kidney Vero E6 cell line (kindly gifted by Spallanzani Institute, Rome, Italy) and Calu-3 lung epithelial cell lines (ATCC HTB-55) were maintained in Dulbecco’s Modified Eagle Medium (DMEM; Gibco, Thermo Fisher Scientific, Waltham, MA, USA) supplemented with 10% fetal bovine serum (FBS; Gibco, Thermo Fisher Scientific, Waltham, MA, USA) at 37 °C in a humidified atmosphere of 5% CO_2_.

### 4.2. VSV_Pseudo SARS-CoV-2 Omicron B.1.1.529, BA.4/5, BQ.1.1, and XBB.1.5 Strains Spike with Luciferase Reporter

For cell infection, the VSV_Pseudo SARS-CoV-2 S was used. This pseudotyped virus uses recombinant vesicular stomatitis virus (Rvsv) to carry the spike protein of SARS-CoV-2 (GenBank:MN908947), with multiple mutations initially identified in the Omicron BA.4/5, BQ.1.1, and XBB.1.5 variants. The pseudovirus infectivity of rVSV without its original G is restricted to a single round of replication, and infection of cells with these pseudotyped virus carrying luciferase reporter results in high level luciferase activity.

### 4.3. Protein Preparation of RBD Variants and Docking Simulations

All computational studies were carried out using Schrödinger Suite 2018-1 [40]. The X-ray crystallographic structures of SARS-CoV-2 Omicron variants such as B.1.1.529 (PDB code:7WPB) [41] and BA.4/5 (PDB code:7XWA) [42] were used as receptors. For each investigated variant, the RBD domain (333-527) was extracted and prepared using the Maestro Protein Preparation Wizard tool [43] with OPLS_2005 [44] as a force field at pH 7.4. The RBD structures were optimized by the addition of missing loops using Prime software (ver. 2.1), and the protonation state of the ionizable amino acid residues was determined using the Epik program [45]. Regarding the Omicron sub-variants such as BQ.1.1 and XBB.1.5, the mutated models were generated via single-residue replacement, and the obtained structures were submitted to 10,000 MacroModel minimization steps using OPLS_2005 as a force field [44].

### 4.4. Rational Peptides Design against RBD Domain

We used the DPP4-binding helix extracted from the experimental structure of the DPP4 (PDB code: 2G63), as reported in a previous study [19], as the starting point to design novel DPP4 peptide binders that were able to maintain inhibitory activity against SARS-CoV-2, given the emergence of new variants. An in-house script helped us to generate the scrambled peptide sequences of 6-mer, 7-mer, 8-mer, and 9-mer. The three-dimensional structures of these peptides were obtained by using the build_peptide.py script of the Maestro Build Panel within the graphic interface Maestro Schrödinger ver 11.5 [46,47,48]. All the peptides were energy minimized with the OPLS_2005 force field [44] as implemented in the Schrödinger suite, and the lowest-energy conformations were used as the starting points for the docking simulations. SP-peptide modules from Glide [49] were applied to perform docking of the smaller peptide sequences with default parameters. During the molecular recognition computations, the receptor was fixed, while the peptides were kept fully flexible. Three grids were generated, encompassing the entire cavity, suitable for peptide docking. After the identification of the best-docked sequences and their superimposition on the RBD surface, to reconstruct 26-mer peptides like the original DPP4_270–295_ sequence, these segments were concatenated based on their sequence order.

### 4.5. Peptide Design and Production

The DPP4_270–295_ peptide was designed by evaluating those reported in Nianshuang Wang et al. [50], as previously tested [19,51] (spectra 2000s.r.l). The new promising sequence of 26-mer, named *pep6*, was VTNATAPNTDVSVSLSMILQTISASI. The source of *pep6* was NovoPep Limited (Purity (HPLC) ≥95% 95.72%).

### 4.6. In Silico Analysis of the Conformations of DPP4-Derived Peptides

The 3D structures of the DPP4_270–295_ peptide and the designed sequences were built using the Maestro GUI (Schrödinger Suite 2018-1) and submitted to Protein Preparation Wizard [43]. To catch the conformational variability of the peptides, we ran 500 ns of molecular dynamics simulations (MDs) using the Desmond package ver. 4.2 [52]. We used OPLS_2005 as a force field and an orthorhombic box with a TIP3P water model extending 10 Å outside the complex in all sides for considering the water solvent effects on the peptides’ conformational properties. Electroneutrality was ensured by adding appropriate Na^+^Cl^−^ counter ions to each solvation box. The solvated structures were relaxed by using the Martyna-Tobias–Klein isobaric–isothermal ensemble (MTK_NPT). MDs were carried out using the following conditions: the NPT ensemble, a constant temperature of 300 K, a pressure of 1 bar, and a recording interval equal to 100 ps both for energy, and for trajectory, collecting 1000 frames for each simulation. The trajectory coordinates were submitted to a clustering analysis by using single-linkage method, and for each peptide, the best representative conformation was docked into the RBD binding region for each mutant.

Knowledge-based protein–protein docking of RBD and DPP4_270–295_ and the designed peptides was performed using the HADDOCK 2.4 (High Ambiguity-Driven biomolecular DOCKing) web-server [53]. This tool combines Coulomb electrostatic energies, non-bonded intermolecular Van der Waals forces, and empirically derived desolvation energies and buried surface areas. RBD amino acids exposed to the surface at positions 446, 472, 474, 484, 490, 497, 499, and 502 were considered as active residues, while passive residues were automatically identified as residues surrounding the active ones before submitting the docking job. Regarding the designed peptides, all amino acids were considered as active residues. For each complex, the sampling parameters were as follows: 1000 structures for rigid-body docking, 200 structures for the final refinement, and a cut-off equal to 5.0 to define neighboring flexible regions. For each complex, the docked structures were loaded in the Maestro interface of the Schrödinger Suite 2018-1 in PDB format for visual inspection.

Hotspot residues at the binding interface between the analyzed peptides and the RBD of the BA4.5 and BQ.1.1 variants were identified using GBPM analysis [20]. As reported in previous studies [10,54,55], in order to evidence hydrophobic, hydrogen bond donor and acceptor spots, we used DRY, N1, and O GRID probes, respectively. For each complex, RBD was seen as a guest, while the investigated peptides were hosts. The selected residues at the interface covered a maximum distance of 3 Å from the most-relevant interaction energy points (GBPM features) of the computed molecular interaction fields (MIFs). After selecting an energy cut-off that was 30% above the global minimum, the pivotal hotpots resulted from the summa of the related GBPM features’ interaction energy.

### 4.7. MTS and In Vitro Neutralization Assay of DPP4_270–295_ and DPP4-Derived Peptide

For the MTS assay, VERO E6 cells (2 × 104/well) were seeded into 96-well plates in triplicate and cultured in growth medium. Then, different peptide concentrations were added, and the viability was tested every day for three consecutive days using the CellTiter 9 Aqueous One Solution Cell Proliferation Assay kit (CTB169; Promega, Fitchburg, WI, USA) following the manufacturers manual. Untreated cells were used for value normalizations.

For neutralization experiments with SARS-CoV-2 VSV-based pseudoparticles, Vero E6 and Calu-3 cells were seeded in 96-well plates in 100 μL of minimal essential medium Eagle at 20,000 cells/well. The next day, both diluted DPP4_270–295_ and *pep6* peptides were used at the same concentration of 100 µg, as previously reported [17,19,39], in PBS and added to SARS-CoV-2 VSV-based pseudoparticles CoV2S-PPs at a 1:1 dilution with a maximum of 2% serum. The DPP4/pseudovirus mixtures were incubated for 1 h at 37 °C and then added to the Vero E6 cells/Calu-3 in triplicate. The cells were incubated at 37 °C and 5% CO_2_. Then, 48 h post-infection, Firefly luciferase activity was measured using the Promega Luciferase Assay System (E1501).

### 4.8. Surface Plasmon Resonance (SPR) Assay

SPR measurements were conducted using a Biacore X100 device (Cytiva, Washington, DC, USA) at 25 °C in order to characterize the interaction of the DPP4_270–290_ and *pep6* with the SARS-CoV-2 Omicron BA.5 sublineage receptor binding domain (RBD) immobilized on a CM5 sensor chip. For this aim, 20 µg/mL of RBD of SARS-CoV-2 belonging to Omicron BA.5 (R&D systems, Minneapolis, MN, USA) was diluted in 0.01 M Hepes, 0.05 mM EDTA, and 0.005% surfactant P20, pH 7.4 (running buffer) and injected over the CM5 sensor chip surface at a flow rate of 5 ul/min, allowing for the immobilization of 2100 resonance units (RUs), equal to 0.08 pmol/mm^2^ of Omicron BA.5 RBD. An empty sensor chip was used to evaluate nonspecific binding and for blank subtraction. DPP4_270–290_ and *pep6* at different concentrations (ranging from 9.375 to 150 µg/mL) were diluted in running buffer and injected over the Omicron BA.5 RBD surface for 2 min, then washed until dissociation. After each run, the sensor chip was regenerated via injection of 10 mM of glycine buffer pH = 1.5. The affinity values were calculated using the nonlinear fitting single-site model software package BIAevaluation (version 3.2 [Cytiva]).

### 4.9. Gene Expression Analysis

The total RNA was extracted from Calu-3 cells using TRIzol (Ambion, Foster City, CA, USA) according to the manufacturer’s instructions, followed by reverse transcription (RT) of 1 μg of RNA using the Life Technologies Corporation’s High-Capacity cDNA Archive kit (Foster City, CA, USA). An expression analysis was performed using quantitative RT-polymerase chain reaction (SYBR Green Assay; Applied Biosystems Inc., Foster City, CA, USA) using the 7500 Real-Time PCR System (Applied Biosystems Inc., Foster City, CA, USA). Primer sequences will be given upon request. We chose GAPDH as the internal reference gene. The 2^-DCt^ and comparative DDCt methods were used to quantify the relative gene expression levels.

### 4.10. Quantification and Statistical Analysis

All the experiments were performed in technical duplicates, and the data were analyzed using GraphPad Prism 8 and the SPSS program, version 25 (IBM Corp, Armonk, NY, USA).

## 5. Conclusions

To date, other variants derived from Omicron are emerging. Our study outlines a proof of principle, indicating that the peptide-based technology represents a strategy that can dynamically and promptly counteract the viral infection, as it is possible to adapt weapons to fight it properly (by in silico modifying and adapting the peptide sequence). The researchers maintain that real-time monitoring of pathogens requires interoperability and interconnectivity between genomic, clinical, and epidemiologic surveillance systems. For clinicians, it means ‘precision public health’ alerts will provide clues for patient care to improve patient outcomes as well as help prevent epidemic spread in the community [56]. The development of pan-coronavirus inhibitors and their combinations, ideally delivered orally or by inhalation, could be useful in fighting SARS-CoV-2 variants, and the availability of such therapies would be highly attractive in preparation for future outbreaks of pathogenic coronaviruses. Effective treatment strategies that can be conveniently applied are still needed to tackle COVID-19, including those that can address the drug resistance conferred by emerging variants. Although this study has some limitations, including not having used the live virus, our results both in silico and in vitro allowed us to identify a promising peptide that is capable of binding to the SARS-CoV-2 RBD, despite the emergence of new variants.

To limit the initial pathogenicity of a pandemic agent, while awaiting the production of an effective vaccine, the use of rationally selected site-specific synthetic polypeptides may be useful. The strategy is to identify the cellular receptor binding domains that the infectious agent uses to enter the target cells, then synthesize a polypeptide (26 aa in the present work) that simulates these reactive domains with the key molecules of the pathogen. Variants on the pivotal infectious protein typically modify immune-dominant antibody epitopes without significantly changing cellular receptor binding affinity. Significant variations are unlikely to occur in the pathogen molecule’s binding site with the cell membrane, as they would modify the infecting capacity. The reaction of the polypeptide with the key molecule of the pathogen inhibits and/or reduces the infecting capacity, with effects on the concentration and speed of diffusion pending the production of an effective vaccine. These findings could lead to the development of a valuable modality for the prophylactic or therapeutic treatment of COVID-19.

## Figures and Tables

**Table 1:** Comparison between the representative cluster of DPP4_270–295_ after molecular recognition with RBD conformation of B.1.1.529, BA.4/5, and BQ.1.1 variants using the HADDOCK tool.

	HADDOCK Score *	Cluster Size	RMSD **	VdW Energy	Electrostatic Energy	Desolvation Energy	BSA ***	Z-Score ****
RBD B.1.1.529	−80.6 ± 2.8	39	3.2 ± 0.1	−53.6 ± 2.6	−87.1 ± 11.3	−17.1 ± 2.1	1577.5 ± 35.4	−1.9
RBD BA.4/5	−74.4 ± 2.9	50	3.6 ± 0.1	−63.0 ± 2.0	−44.2 ± 7.4	−17.0 ± 3.4	1586.7 ± 76.0	−2.5
RBD BQ.1.1	−77.7 ± 2.9	20	3.8 ± 0.1	−55.3 ± 3.0	−95.1 ± 21.0	−9.3 ± 2.0	1718.3 ± 94.2	−1.6

* The HADDOCK score is defined as 1.0 van der Waals intermolecular energy (Evdw) + 0.2 electrostatic intermolecular energy (Eelec) + 1.0 desolvation energy (Edesol) + 0.1 distance restraints ambiguous energy (EAIR). ** Root mean square deviations (RMSD) from the overall lowest-energy structure. *** Buried surface area. **** HADDOCK Z-score indicates standard deviations from the average cluster (the more negative, the better).

**Table 2:** GBPM average scores and quartile distributions of the pivotal hotspots of DPP4_270–295_ and *pep2* in complex with RBD BA4.5 and BQ1.1.

	DPP4_270–295_–RBD BA4.5		DPP4_270–295_–RBD BQ.1.1	
Residue	Total Score	Quartile	Total Score	Quartile
T4	−3.31	Q3	-	-
D5	−17.17	Q1	−6.79	Q2
L7	-	-	−4.92	Q4
S9	−8.55	Q1	-	-
V10	-	-	−10.98	Q1
T11	−1.93	Q4	−20.06	Q1
N12	−0.94	Q4	-	-
A13	−5.20	Q2	-	-
T14	−8.39	Q2	-	-
I16	−4.82	Q2	-	-
Q17	−34.50	Q1	−12.11	Q1
I18	-	.	−5.43	Q3
A22	−1.73	Q4	−3.20	Q4
S23	−2.86	Q3	−5.46	Q2
M24	-	-	−5.16	Q3
	***Pep2*–RBD BA4.5**		***Pep2*–RBD BQ.1.1**	
**Residue**	**Total Score**	**Quartile**	**Total Score**	**Quartile**
V1	−18.55	Q1	-	-
T2	−0.76	Q4	-	-
Q8	−21.99	Q1	-	-
I9	−5.78	Q3	-	-
N12	−5.80	Q2	-	-
T13	−17.52	Q2	-	-
D14	-	-	−22.57	Q1
S15	-	-	−14.58	Q1
L16	−0.52	Q4	-	-
S17	-	-	−7.23	Q3
S18	-	-	−8.47	Q3
T19	-	-	−10.99	Q2
A22	-	-	−0.24	Q4
L25	-	-	−1.76	Q4
I26	-	-	−12.22	Q2

**Table 3:** Comparison between the representative cluster of *pep6* after molecular recognition with RBD conformation of BA.4/5, BQ.1.1, and XBB.1.5 variants using the HADDOCK tool.

	HADDOCK Score *	Cluster Size	RMSD **	VdW Energy	Electrostatic Energy	Desolvation Energy	BSA ***	Z-Score ****
RBD BA.4/5	−97.1 ± 2.4	56	0.4 ± 0.2	−56.3 ± 4.1	−208.6 ± 35.1	−10.2 ± 1.7	1522.2 ± 41.9	−2.4
RBD BQ.1.1	−91.3 ± 1.1	31	0.4 ± 0.3	−61.6 ± 2.9	−102.2 ± 23.0	−16.8 ± 2.1	1669.1 ± 36.0	−2.6
RBD XBB.1.5	−91.7 ± 2.7	37	0.7 ± 0.4	−61.6 ± 6.2	−126.2 ± 14.9	−11.4 ± 5.3	1702.5 ± 37.4	−2.3

* The HADDOCK score is defined as 1.0 van der Waals intermolecular energy (Evdw) + 0.2 electrostatic intermolecular energy (Eelec) + 1.0 desolvation energy (Edesol) + 0.1 distance restraints ambiguous energy (EAIR). ** Root mean square deviations (RMSD) from the overall lowest-energy structure. *** Buried surface area. **** HADDOCK Z-score indicates standard deviations from the average cluster (the more negative, the better).

## Data Availability

All data generated or analyzed during this study are included in this published article and its Supplementary Marterials Files.

## Supplementary Materials

The following supporting information can be downloaded at: https://www.mdpi.com/article/10.3390/ph17070891/s1, Table S1. Full sequence of peptides obtained by combining three fragments, named seq1 (VVNTDSLSS), seq2 (VTNATSIQI), and seq3 (TAPASMLI), derived by the splitting of the original peptide (DPP4_270–295_); Table S2. Comparison between the representative cluster of 5 query sequences (Pep1–Pep5) after molecular recognition with RBD conformation of BA.4/5 and BQ.1.1 variants using the HADDOCK tool; Table S3. Glide docking score (G-Score) values of the 15 best docked peptide sequences against the RBD surface. The G-score values are reported in kcal/mol; Table S4. Concatenation of the best-docked sequences of peptides of 6-mer, 7-mer, 8-mer, and 9-mer on the BA.4/5 RBD binding pocket; Source code for the generation of random sequences of 6-mer, 7-mer, 8-mer-, and 9-mer.
