# Supplementary material for: A Dynamic and Effective Peptide-Based Strategy for Promptly Addressing Emerging SARS-CoV-2 Variants of Concern
Source: Pharmaceuticals (Basel). 2024 Jul 4;17(7):891. doi: 10.3390/ph17070891 (PMC11279616; doi:10.3390/ph17070891)
Supplement: Supplementary file 1 [file pharmaceuticals-17-00891-s001.zip › pharmaceuticals-3043541-supplementary.pdf]

Supplementary Materials

A Dynamic and Effective Peptide-Based Strategy for Promptly Addressing Emerging SARS-CoV-2 Variants of Concern

Michela Murdocca [1,†], Isabella Romeo [2,3,†], Gennaro Citro [1], Andrea Latini [1], Federica Centofanti [1], Antonella Bugatti [4], Francesca Caccuri [4], Arnaldo Caruso [4], Francesco Ortuso [2,3], Stefano Alcaro [2,3], Federica Sangiuolo [1,*] and Giuseppe Novelli [1,5,6]

[1] Department of Biomedicine and Prevention, University of Rome Tor Vergata, Via Montpellier 1, 00133 Rome, Italy

[2] Dipartimento di Scienze della Salute, Università "Magna Græcia" di Catanzaro, Campus "S. Venuta", Viale Europa, 88100 Catanzaro, Italy

[3] Net4Science Srl Academic Spin-off, Università "Magna Græcia" di Catanzaro, Campus "S. Venuta", Viale Europa, 88100 Catanzaro, Italy

[4] Section of Microbiology, Department of Molecular and Translational Medicine, University of Brescia, 25123 Brescia, Italy

[5] IRCCS Neuromed Mediterranean Neurological Institute, 86077 Pozzilli, Italy

[6] Department of Pharmacology, School of Medicine, University of Nevada, Reno, NV 89557, USA

* Correspondence: sangiuolo@med.uniroma2.it

† These authors contributed equally to this work

Sommario

Table S1. Full sequence of peptides obtained by combining three fragments, named *seq1* (VVNTDSLSS), *seq2* (VTNATSIQI) and *seq3* (TAPASMLI), derived by the splitting of the original peptide (DPP4$_{270-295}$).

Table S2. Comparison between the representative cluster of 5 query sequences (*Pep1-Pep5*) after molecular recognition with RBD conformation of BA.4/5 and BQ.1.1 variants through the HADDOCK tool.

Table S3. Glide docking score (G-Score) values of 15 best docked peptide sequences against RBD surface. The G-score values are reported in kcal/mol.

Table S4. Concatenation of the best docked sequences of peptides of 6-mer, 7-mer, 8-mer, and 9-mer on BA.4/5 RBD binding pocket.

Source code for the generation of random sequences of 6-mer, 7-mer, 8-mer- and 9-mer.

Table S1. Full sequence of peptides obtained by combining three fragments, named *seq1* (VVNTDSLSS), *seq2* (VTNATSIQI) and *seq3* (TAPASMLI), derived by the splitting of the native peptide (DPP4$_{270-295}$).

**Peptide_name**	**Sequence_combination**	**Full_sequence**
DPP4$_{270-295}$	seq1_seq2_seq3	VVNTDSLSSVTNATSIQITAPASMLI
Pep1	seq1_seq3_seq2	VVNTDSLSSTAPASMLIVTNATSIQI
Pep2	seq2_seq1_seq3	VTNATSIQIVVNTDSLSSTAPASMLI
Pep3	seq2_seq3_seq1	VTNATSIQITAPASMLIVVNTDSLSS
Pep4	seq3_seq1_seq2	TAPASMLIVVNTDSLSSVTNATSIQI
Pep5	seq3_seq2_seq1	TAPASMLIVTNATSIQIVVNTDSLSS

Table S2. Comparison between the representative cluster of 5 query sequences (*Pep1*-*Pep5*) after molecular recognition with RBD conformation of BA.4/5 and BQ.1.1 variants through the HADDOCK tool.

	HADDOCK score	Cluster size	RMSD	VdW energy	Electrostatic energy	Desolvation energy	BSA	Z-score
Pep1 BA.4/5	-80.2 +/- 7.3	10	0.8 +/- 0.6	-54.0 +/- 8.1	-86.4 +/- 15.5	-20.1 +/- 1.1	1645.3 +/- 98.4	-2.3
Pep2 BA.4/5	**-84.2** +/-3.0	14	12.0 +/- 0.2	-57.4 +/- 4.3	-128.0 +/- 24.4	-7.6 +/- 1.9	1548.2 +/- 47.4	-1.3
Pep3 BA.4/5	-78.0 +/- 3.6	11	8.3 +/- 0.0	-58.1 +/- 3.9	-85.3 +/- 11.6	-16.9 +/- 1.3	1563.4 +/- 52.3	-2.1
Pep4 BA.4/5	-66.8 +/- 1.2	27	3.9 +/- 0.2	-53.8 +/- 18.1	-58.6 +/- 18.1	-16.6 +/- 2.0	1368.2 +/- 57.2	-1.6
Pep5 BA.4/5	-62.0 +/- 5.1	16	0.5 +/- 0.4	-53.8 +/- 6.8	-35.2 +/- 13.7	-19.0 +/- 1.9	1433.6 +/- 71.3	-1.7
Pep2 BQ.1.1	-72.1 +/- 10.4	10	1.0 +/- 0.6	-44.7 +/-6.8	-162.4 +/- 16.4	-9.6 +/- 3.4	1356.7 +/- 22.8	-2.0

Table S3. Glide docking score (G-Score) values of 15 best docked peptide sequences against RBD surface. The G-score values are reported in kcal/mol.

Sequence	Gscore value
VTNATA	-5.82
TVNATA	-5.78
NVTATA	-5.75
QTISASI	-6.54
QSTISIA	-7.05
QSITISA	-6.81
APNDVTSV	-9.10
APNTDVSV	-9.81
APNVDTSV	-9.82
NTDSVLSVS	-8.74
NSTVVLSDS	-8.34
NTDVSVSLS	-8.29
SLSMILQT	-6.23
SLSLMITQ	-6.31
SLSMILTQ	-6.42

Table S4. Concatenation of the best docked sequences of peptides of 6-mer, 7-mer, 8-mer, and 9-mer on BA.4/5 RBD binding pocket.

Best docked sequences					26-mer concatenated sequences
VTNAT**A**	**A**PNDVTSV				
	**A**P**NTDVSV**	**NTDVSVSLS**	**SLS**MIL**QT**	**QT**ISASI	VTNAT**APNTDVSVSLS**MIL**QT**ISASI
				QSTISIA	
				QSITISA	
			**SLS**LMITQ		
			**SLS**MILTQ		
	**A**PNVDTSV				
TVNAT**A**	**A**PNDVTSV				
	**A**P**NTDVSV**	**NTDVSVSLS**	**SLS**MIL**QT**	**QT**ISASI	TVNAT**APNTDVSVSLS**MIL**QT**ISASI
				QSTISIA	
				QSITISA	
			**SLS**LMITQ		
			**SLS**MILTQ		
	**A**PNVDTSV				
NVTAT**A**	**A**PNDVTSV				
	**A**P**NTDVSV**	**NTDVSVSLS**	**SLS**MIL**QT**	**QT**ISASI	NVTAT**APNTDVSVSLS**MIL**QT**ISASI
				QSTISIA	
				QSITISA	
	**A**PNVDTSV		**SLS**LMITQ		
			**SLS**MILTQ		

Source code for the generation of random sequences of 6-mer, 7-mer, 8-mer- and 9-mer.

```
import itertools
# Define the amino acid sequence
sequence = "DLNSSSTVV"
sequence = "AANTVV"
sequence = "AIIQSST"
sequence = "ADNPSTVV"
sequence = "ILMQSST"
# Generate all permutations of the sequence
permutations = set(itertools.permutations(sequence))
# Convert permutations to strings and sort them
peptide_list = [''.join(p) for p in permutations]
peptide_list.sort()
# Write the list of scrambled peptides to a file with open('scrambled_peptides.txt', 'w') as file:
    for peptide in peptide_list:
        file.write(peptide + '\n')
```
